# Core components of end-of-life care in nursing education programs: a scoping review

**DOI:** 10.1186/s12904-024-01398-3

**Published:** 2024-03-28

**Authors:** Zahra Taheri-Ezbarami, Fateme Jafaraghaee, Ali Karimian Sighlani, Seyed Kazem Mousavi

**Affiliations:** 1 Department of Nursing, School of Nursing and Midwifery, Guilan University of Medical Sciences, Rasht, Iran; 2 Department of Philosophy, Guilan University, Rasht, Iran; 3 Department of Nursing, Abhar School of Nursing, Zanjan University of Medical Sciences, Zanjan, Iran; 4 Department of Nursing, School of Nursing and Midwifery, Guilan University of Medical Sciences, Rasht, Iran

**Keywords:** End of life care, Palliative care, Program, Nurses

## Abstract

**Background:**

So far, there have been many studies on end-of-life nursing care education around the world, and in many cases, according to the cultural, social, and spiritual contexts of each country, the results have been different. The present study intends to gain general insight into the main components of end-of-life care in nursing education programs by reviewing scientific texts and the results of investigations.

**Methods:**

This study was a scoping review conducted with the Arksey and O’Malley methodology updated by Peters et al. First, a search was made in Wos, ProQuest, Scopus, PubMed, Science Direct, Research Gate, and Google Scholar databases to find studies about end-of-life care education programs. Then, the screening of the found studies was done in four stages, and the final articles were selected based on the inclusion and exclusion criteria of the studies. Due to the nature of the research, editorials, letters, and commentaries were excluded. The screening steps are shown in the PRISMA-ScR diagram.

**Results:**

23 articles related to end-of-life care education programs were reviewed. The studies included eleven descriptive and cross-sectional studies, two qualitative studies, eight interventional studies, one concept analysis article, and one longitudinal study. By summarizing the data from the studies, six themes were obtained as the main components of end-of-life care education: principles of end-of-life care, communication skills, physical considerations, psychosocial and spiritual considerations, ethical considerations, and after-death care.

**Conclusion:**

End-of-life care is one of the most challenging nursing care in the world. Since many nurses are not prepared to provide such care, the information obtained from this review can help nursing education and treatment managers develop more comprehensive training programs to improve the quality of end-of-life care.

## Background

Death, as an inseparable aspect of human existence, has been the subject of everyone’s concern for many years [1]. This process is a bio-psychosocial and spiritual experience that, despite significant advances in medical science and technology, there is still no way to avoid it [2]. Today, due to the increase in life expectancy and relative well-being, more people are treated in different clinical environments in the final stages of life, and most deaths occur in conditions where patients are often isolated and under mechanical ventilation. Therefore, their families are concerned about how to care for them in such environments [3]. In this period, the needs and requests of patients and companions and the stressfulness of the conditions cause avoidance of participation in care decisions, dissatisfaction of care workers, disregard for care details, and, as a result, decreased quality of care [4].

End-of-life care is a part of palliative care and includes the care of people whose disease has progressed and are approaching the end of their lives [5]. Depending on the nature of the disease, this type of care can be provided in the last year, month, week, day, and hours of a person’s life [4]. Therefore, its main difference from palliative care is that palliative care can be used at any point in the treatment process. However, end-of-life care is at the end of the treatment process and is given to people with advanced, progressive, and incurable diseases [5]. It helps them live in the best possible way when death arrives [6]. Like palliative care, end-of-life care advocates the idea of a holistic, comprehensive and patient-centred, thus often includes a wide range of interventions and dimensions such as physical, emotional, mental, psychological, spiritual and social [7]. . By performing this type of care, patients’ and their families’ support and palliative needs are identified and answered in their last life stage [8]. Usually, these cares are provided in an environment filled with emotional and moral challenges. Therefore, issues related to end-of-life care are known as one of the world’s ten most significant ethical challenges, and receiving sustainable end-of-life care with the best quality has become one of policymakers’ and health managers’ most important concerns [4, 7].

According to the available statistics, every year in the world, about 55 million people need palliative care, and 25 million people need end-of-life care [9]. Meanwhile, as the largest group of health workers, nurses play the most essential role in end-of-life care [5]. Caring for a dying patient and comforting and comforting his family is one of the most challenging nursing experiences [10]. Nurses can have the last and most significant effects on the person’s way of life until the time of death, the customs related to the event, and the family’s final memories of death [11]. However, the findings show that most nurses, especially those working in developing countries, need more capabilities in providing palliative and end-of-life care [8, 12]. Since most body systems are affected in the final stages of life and show abnormal performance, nursing care interventions in these patients are complex and require high theoretical knowledge and practical skills. This is even though many nurses do not receive such training in full while studying and working [5, 13]. Failure to provide palliative and end-of-life education for nurses is not new, and many reputable nursing organizations call it a historical flaw in the nursing education program [14].

Even though more than half a century has passed since the introduction of end-of-life care into the nursing curriculum, studies show that in most countries, there are severe deficiencies in quantity and quality regarding the education of this care, especially clinically [15]. For example, the findings of a study showed that out of three million working nurses in the United States, only 600,000 of them had completed the end-of-life care course, and less than 40% had such content in the program during their education [16]. So far, various studies have been conducted regarding end-of-life care education for nurses and nursing students in clinical and academic settings. However, the void of a comprehensive study categorizing these scattered findings and providing a solid theoretical foundation for future research is felt. Therefore, the authors decided to review and organize the main components of end-of-life care in all undergraduate and graduate nursing studies, regardless of the educational environment and type of education (theoretical or clinical).

The present study was planned and implemented with this aim.

## Methods

A scoping review is used to quickly review key concepts in a specific research topic and find the primary sources and types of available evidence. This method can be project-specific, especially for complex issues not comprehensively reviewed [17]. In 2005, for the first time, Arksey and O’Malley presented a framework for conducting a scoping review, which is considered and used as the main guideline of this research methodology [18]. In this study, the steps proposed by Peters et al., the updated framework version, have been used [19].

### Step 1: title and review questions

A preliminary search of the peer-reviewed primary research studies helped refine the research questions. The research questions aimed to determine the main components of end-of-life care in nursing education programs. These questions were: (1) Are there any suitable studies regarding end-of-life care education programs in nursing? (2) What are the main components of these educational programs implemented for nurses and nursing students?

### Step 2: inclusion criteria

The inclusion criteria included electronic studies on end-of-life care education for nurses and nursing students published in English between 2015 and 2023.

### Step 3: search strategy

The search was conducted to find studies using standard keywords in Wos, ProQuest, Scopus, PubMed, Science Direct, Research Gate, and Google Scholar. The keywords were used individually or with the prepositions “AND” and “OR” based on Boolean Logic. The combination of keywords used is shown in Table 1.

**Table 1:** Combination keywords used in all databases

“End-of-life care” OR “End of life care” OR “Dying care” OR “Hospice care” OR “Overseeing death care” OR “Near death care” OR “Death care” OR “Care at the time of death” OR “Care when dying” OR “Terminal Care” OR “Palliative care” OR “Comforting care” OR “Comprehensive care” OR “Respectful care” OR “Hospice and Palliative Care” OR “Hospice and Palliative Care Nursing” OR “Advance Care Planning” OR “Long-Term Care” OR “Critical Care Nursing” OR “Nursing Care” OR “Critical Care” OR “Patient Care” OR “Comprehensive Health Care” AND “Curriculum” OR “ Nursing curriculum” OR “Program” OR “Program Development” OR “Nursing program” OR“ Education” OR “Educational program” OR “Educational content” OR “Educational components” AND “Nursing” OR “Nursing student(s)” OR “Student(s), OR “Nurse(s)”	

### Step 4: evidence screening and selection

**Fig. 1:**
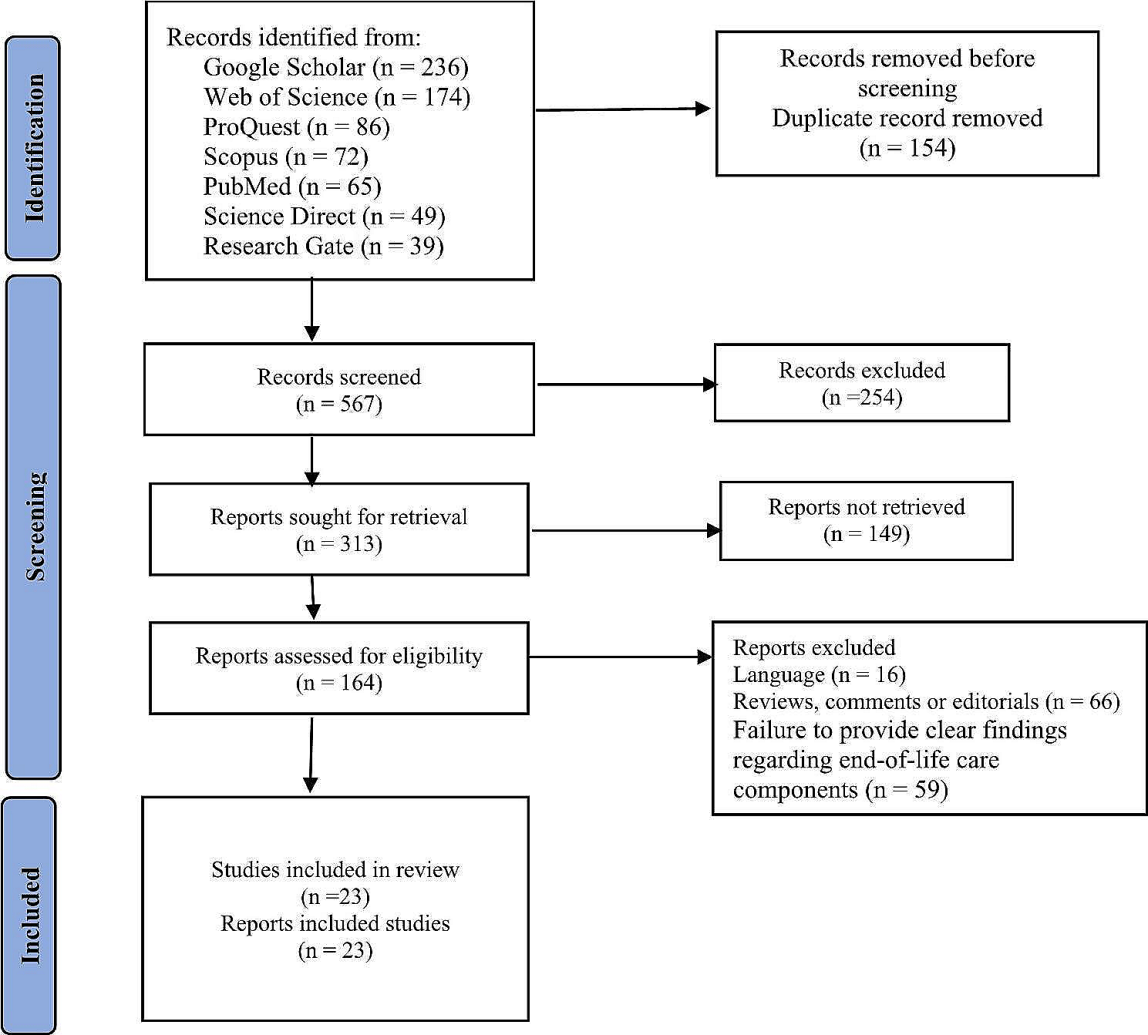
PRISMA-ScR diagram of screening process and selection of articles

Based on the search strategy, 721 articles were initially obtained. Then, the studies were screened in four stages based on the inclusion criteria by two-channel teams, including researchers and an experienced research librarian (to avoid bias). In the first screening, articles unrelated to end-of-life care education programs were excluded; duplicate reports were removed in the second step. In the third screening, editorials, letters, and commentaries were excluded due to the nature of the research. In the fourth and last screening stage, articles related to the study’s title that did not provide clear findings about end-of-life care components were excluded from the study. Finally, 23 articles were included in the study. The study selection process, reported in a flow diagram, as proposed by the PRISMA-ScR diagram (Fig. 1).

### Step 5: data extraction

The data obtained from the studies were summarized in a table (Table 2) and written separately for each survey. These data include the author’s name (s), year of publication, country, purpose, sample size, research method, and key findings.

**Table 2:** Summary of studies included in the scoping review

Author(s)	Year of publication	Country	Research goal	Sample size	Research method	Key findings
La Sala et al. [20]	2023	Italy	Education nursing students’ in palliative care and pain therapy	109 nursing students of a college in Parma city	Descriptive	According to the questionnaire, the six areas of fear management, helping the patient’s family, improving communication, strengthening the caring role of the family, improving the relationship with the patient, and helping to meet the patient’s personal needs were considered educational topics in this study.
Jeong et al. [11]	2023	South Korea	Implementation and evaluation of an end-of-life care education program for oncology nurses	70 nurses of the oncology department	Quasi experimental	The educational content of this program included good death, sharing experiences regarding end-of-life care, principles of end-of-life care, and communication skills.
Ghaemizade Shushtari et al. [8]	2022	Iran	Effect of end-of-life nursing education on the knowledge and performance of nurses in the intensive care unit	80 nurses working in the intensive care units of two hospitals	Quasi experimental	The educational content was based on the nine main topics of end-of-life care. It included principles of end-of-life nursing care, pain management, symptom management, ethical issues, cultural and spiritual considerations, communication, grief and bereavement, access to care with Quality of life, and dying care.
Wong et al. [12]	2022	Hong Kong, China	Effectiveness of educational programs on palliative and end-of-life care in promoting perceived competence among health and social care professionals	779 (physicians, nurses and social workers)	Descriptive	The educational content of these programs includes: (1) Values and knowledge (2) Communication skills (3) Management of symptoms (4) Psychosocial care (5) Deciding on end-of-life care (6) Grief (7) self-care.
Kennedy et al. [21]	2022	Ireland	Reimagining a children’s palliative care educational programme for registered nurses in response to the COVID-19 pandemic	169 nurses	Descriptive- correlational	The educational content included: (1) An introduction to palliative care for children (2) Caring for a child with life-threatening conditions (3) Management of pain and physical symptoms (4) Social support (5) Psychological support (6) Joint decision-making regarding end-of-life issues
Wang et al. [22]	2022	Macau, China	Nursing undergraduates’ experiences of a simulation-centred educational program in hospice care	17 undergraduate nursing students	Descriptive, Qualitative	Examining the content of the students’ interviews showed two themes and six sub-themes: 1) developing students’ competencies in caring for dying patients and their families (sensitivity to patients’ needs, improving knowledge of caring for dying patients, skills in controlling symptoms and providing comfort, Communication skills). 2. Improving the ability to self-care and support colleagues (reflection on life and death, encouraging sharing of feelings and support among colleagues).
Ozturk Birge et al. [23]	2021	Turkey	Effect of Education Given to Nursing Students on Their Palliative Care Knowledge and Attitudes	105 s year nursing students	One group pre and post-test	The educational content designed in this study included the following items: (1) Defining palliative care, stating its goals, and stating the history; (2) Principles of palliative care (3) Duties of the palliative care team and nurse (4) Patient acceptance criteria for palliative care (5) Ethical principles and obstacles to providing palliative care (6) Management of physical symptoms in palliative care (7) Improving communication with the patient and family (8) Preparation for mourning
Mota-Romero et al. [6]	2021	Spain	Nursing homes end of life care program	54 nurses	Descriptive	The content of the program of this study was: (1) General aspects of palliative care (2) Principles of symptom control and convenient care (3) Management of nutrition, excretion, activity and cognitive symptoms (4) Psychosocial care (5) After-death care (6) Communication and decision-making (7) Mourning
Hao et al. [4]	2021	China	Effectiveness of end-of-life educational intervention on nurses’ attitudes and knowledge	97 nurses	Quasi experimental	The three main parts of this educational intervention were: (1) Death and life (life, death, death with dignity) (2) Nursing palliative care (3) mourning
Heath et al. [24]	2021	New Zealand	Preparing nurses for palliative and end of life care	Managers of 13 nursing education institutes	Descriptive - cross-sectional	The educational content included the definition and philosophy of palliative care, attitude to death and dying, assessment and management of pain, shortness of breath, vomiting, constipation, confusion and restlessness, nutrition, bereavement, the impact of illness on the family, spirituality, moral issues, and private.
Eltaybani et al. [3]	2020	Egypt	Palliative and end-of-life care education in nursing curricula	95 nursing instructors	Cross-sectional	The educational content of palliative and end-of-life care considered by the researchers in this study includes the end-of-life process, physical aspects, psychiatric aspects, social dimension, spiritual, religious, and existential dimensions, cultural dimension, patient care at the end of life, and ethical and legal aspects.
Dobrowolska et al. [25]	2019	Poland	Predicted difficulties, educational needs, and interest in working in end of life care among nursing and medical students	112 nursing students and 101 medical students	Cross-sectional	Subjects suggested by both groups to be included in the educational program: (1) How to communicate with the dying patient and his family; (2) Psychological support for the patient and family (3) Physical care of the patient (4) Spiritual and social care of the patient (5) Using new methods for education instead of relying on traditional approaches
Tamaki et al. [26]	2019	Japan	Effectiveness of end-of-life care simulation in undergraduate nursing education	38 third-year undergraduate nursing students	Randomized controlled trial	The education content included the following topics: (1) Pain management (2) Effective therapeutic communication techniques (3) Psycho-emotional support for the patient
Goode et al. [27]	2019	Great Britain	Person-centered end-of-life curriculum design in adult pre-registration undergraduate nurse education	336 undergraduate nursing students	Longitudinal	The main topics of the program were: (1) Preparation for death and dying; (2) Promotion of self-care and self-awareness (3) Preparation for difficult conversations at the end of life (4) Coping skills (5) Supporting the family (6) Considering values
Griffith et al. [13]	2018	Great Britain	Prepared for end-of-life care: a	-	Concept analysis	Some indicators of readiness for end-of-life care were mentioned: 1. The ability to communicate at the desired level, having knowledge about ethical, cultural, and spiritual issues at the end of life, managing emotions, and having empathy skills.
Price et al. [28]	2017	United States of America	Assess nurses’ perceived competency regarding providing palliative and end-of-life care to hospitalized patients.	583 nurses working in intensive care departments for children and adults	Descriptive	According to the questionnaire, the educational needs of nurses included communication, decision-making, symptom management, patient and family support, and staff support. Also, nurses had concerns about improving communication behaviors, decision-making, and facilitating continuous care.
Malik et al. [14]	2017	United States of America	Education End-of-Life Care for Certified Nursing Assistants in Long-Term Care	20 nurses	One group pre and post-test	The topics of educational content included: an overview of palliative nursing care, pain management, care at the time of death, assistance in personal development and self-care, and communication with the patient and family.
Lippe et al. [29]	2017	United States of America	Evaluating End-of-Life Care Education Within Nursing Programs	33 nursing faculty members	Descriptive - cross-sectional	The educational content is the same as the curriculum headings of the end-of-life nursing education consortium (ELNEC)) including an introduction to palliative nursing care, pain management, symptom management, ethical issues in palliative nursing care, cultural considerations in end-of-life care, communication, loss, grief, and bereavement, and final hours.
O’Shea et al. [30]	2017	United States of America	Assessing palliative and end-of-life educational needs of pediatric health care professionals	139 (46 physicians and 93 nurses)	Descriptive	According to the participants of this study, it was more important to include these items in the educational content: (1) Providing patient-centered and family-centered education (2) Considering ethical and cultural issues (3) Training focuses on providing effective care
Jors et al. [31]	2016	Germany	Suggestions from experienced physicians and nurses to improve end-of-life care education	1131 physicians and nurses	Content analysis	The most important suggestions for the content of the program were: teaching the basic principles of palliative care, communication skills, interaction with patient caregivers, pain management, ethical, socio-cultural, spiritual, religious, and psychological issues.
Carman et al. [32]	2016	United States of America	Implementation of a learning bundle to promote end-of-life education for nursing students	71 master’s students	Quasi experimental	The most important components of the training package included the following items: (1) Helping the patient, relatives and colleagues to cope with suffering and grief (2) Attention to the emotional and spiritual needs of the people involved (3) Management of physical symptoms (pain, dyspnea, etc.)
Youssef et al. [33]	2015	Saudi Arabia	Prioritizing palliative care and assessing the adequacy of palliative care content in the nursing curriculum	100 nurses	Cross-sectional	From the perspective of nurses, in teaching palliative care and end-of-life, these things should be prioritized: the purpose of palliative care, pain management, management of other symptoms, communication with the patient and family, paying attention to the role and needs of the patient’s caregivers, death and dying, ethical issues of care end of life.
Murakami et al. [34]	2015	Japan	Development of a neonatal end-of-life care education program for NICU nurses	30 nurses	Quasi experimental	The main elements of the program included an introduction to end-of-life care, ethical decision-making, caring for dying infants, bereavement care for families, communication, and support for nurses.

## Results

### Step 6: data analysis

A total of 23 articles related to end-of-life care education programs were reviewed. The studies included Eleven descriptive and cross-sectional, two qualitative, eight interventional, one concept analysis, and one longitudinal. A list of essential components in end-of-life care education was prepared by gathering the data from the studies. Then, a deep examination of the data and their summarization led to the extraction of codes and, finally, the main themes related to the research question. Therefore, six main themes were obtained as the main components of end-of-life care education: principles of end-of-life care, physical considerations, communication skills, psychosocial and spiritual considerations, ethical considerations, and after-death care (Table 3). It should be noted that the validity of the results was ensured through peer review by two expert faculty members.

**Table 3:** Sub category, Category and Major Themes of end-of-life care components

Major Themes	Category	Sub category
Principles of end-of-life care [4, 11, 14, 22–24, 33, 34]	Definition, objectives and history [23, 24]	Definition of end-of-life care [23, 24]
		A Historical overview of end-of-Life care [23, 24]
	The Process of Dying [4, 11, 23, 33]	Different aspects of death [4, 23, 33]
		Dignified death [4, 11]
	The role of the nurse in end-of-life care [4, 14, 22, 34]	The position of the nurse in end-of-life care [4]
		Essential nursing skills for end-of-life care [14, 22, 34]
Communication skills [11–14, 20, 22, 23, 25, 26, 28, 31, 33, 34].	Principles of effective communication [11, 13, 14, 20, 22, 23, 25, 26, 28, 31, 33, 34]	Patient [11, 14, 20, 22, 23, 25, 28, 33]
		Caregivers [14, 20, 22, 23, 25, 31, 33]
		Healthcare team [11, 13, 26, 34]
	Facilitators and barriers to therapeutic communication with the dying patient [11–13, 22, 28, 29, 34]	Facilitators [11–13, 28, 29, 34]
		Barriers [12, 22, 29]
Physical considerations [6,8,12,14,21,23,24,25,26, 28,29,31,32,33]	Pain management [8, 14, 21, 24, 26, 29, 31–33]	Pharmaceutical treatments [8, 14, 21, 24, 31–33]
		Non-pharmacological treatments [21, 24, 26, 29, 31]
	Nutritional management [6, 24]	Oral nutrition [6, 24]
		Total parenteral nutrition [6]
	Respiratory management [6, 8, 21, 24, 25, 28, 33]	Pharmaceutical treatments [6, 8, 21, 24, 25, 33]
		Non-pharmacological treatments [6, 8, 24, 28]
	Skin care [6, 12, 21, 25, 28, 33]	Prevention of skin problems [6, 25, 28, 33]
		Treatment of skin disorders [12, 21, 25, 28, 33]
	Management of disposal problems [6, 23–25, 29]	Urinary incontinence [6, 29]
		Fecal incontinence [6, 24, 25, 29]
Psychosocial and Spiritual considerations 3,6,8,12,13,20,21,22, 24,25,26,27,29,30,31,32]	Management of Anxiety [3, 6, 12, 21, 25, 26, 31]	Pharmaceutical treatments [3, 6, 12, 31]
		Non-pharmacological treatments [3,31,25,26
	Management of Depression [3, 21, 25, 26]	Pharmaceutical treatments [3, 21]
		Non-pharmacological treatments [3, 25, 26]
	Management of emotional distress [26, 27, 32]	Coping skills [26, 27, 32]
	Patient-centered Care [20, 22, 30]	Ways to maintain maximum patient independence [20, 22, 30]
	Family-centered care [20, 30]	Methods of involving the family in the care process [20, 30]
	Spiritual care [3, 8, 13, 24, 25, 31, 32]	Responding to the spiritual needs [3, 8, 13, 31, 32]
		Responding to the epistemological needs [3, 13, 24, 25]
Ethical considerations [3, 8, 13, 23, 24, 29–31, 33, 34]	Ethical principles [3, 8, 23, 30, 31, 33]	Four ethical principles (Autonomy, beneficence, nonmaleficence, and justice) [3, 8, 23, 30, 31, 33]
	Ethical challenges [3, 8, 13, 24, 29, 30, 34]	Advanced care planning [3, 8, 24, 30]
		Withholding and withdrawing treatment [13, 24, 29, 34]
After-death care [3, 4, 6, 8, 12, 21, 23, 24, 29, 32, 34]	Corpse care [6]	Considerations for the care of the corpse [6]
	Legal considerations [3, 12, 21]	Legal issues after death [3, 12, 21]
	Support the family of the deceased [8, 23, 29]	Improving empathy skills [8, 23, 29]
	Loss and mourning [4, 6, 8, 12, 23, 24, 29, 32, 34]	Management of loss and mourning in the family [4, 6, 8, 12, 23, 24, 29, 32, 34]

### Step 7: presentation of results

#### Theme 1- principles of end-of-life care

In almost all reviewed studies, one of the main components of end-of-life care education programs in nursing is familiarity with the principles of end-of-life care [6, 14, 21, 23, 24, 33, 34]. These studies state that things like definition and historical overview of end-of-life care should be considered at the beginning of educational programs [6, 14, 23, 24]. Jeong et al. in the same context, state that according to many nurses, their position in providing end-of-life care needs to be clarified, and they have challenges in differentiating this type of care from palliative care [11]. Also, according to studies, one of the most important elements of end-of-life care education programs is to explain the concept of death with dignity [4, 11, 27]. Even though more than seventy years have passed since this term was mentioned in the medical literature, many nurses still need clarification in defining this concept [4].

#### Theme 2- communication skills

Communication skills were the main elements of many studies [11–14, 20, 22, 23, 25, 26, 28, 31, 33, 34]. Good communication is essential and challenging to provide good care to a dying person [11, 25]. A person’s needs and preferences can change quickly in their last months, weeks, and days [11]. Good communication is essential to understanding their needs so the healthcare team can meet them [25, 28, 31]. Moreover, it can be challenging to broach the subject of dying within families because no one wants to admit what is coming [20, 22]. However, getting to know communication barriers and facilitators in order to open up the lines of communication and talking about death can go a long way toward relieving anxiety for both parties [14]. In the end, it should be said that effective team communication in the end-of-life stages is necessary to support the patient and the family because it guarantees the transmission of new information and programs at critical times [31, 34].

#### Theme 3- physical considerations

Content related to physical considerations was included in educational programs in about two-thirds of the reviewed studies [6, 8, 12, 14, 21, 23–26, 28, 29, 31–33]. This section included pain management [8, 14, 21, 24, 26, 29, 31–33], control of disposal [6, 23–25, 29] and respiratory [6, 8, 21, 24, 25, 28, 33] status, skincare [6, 12, 21, 25, 28, 33], and improvement of nutritional status [6, 24]. Almost all patients at the end of life suffer from severe pain [8], and many of them have incontinence and skin problems [21, 24, 25]. In addition, dying patients are exposed to malnutrition [24]. On the one hand, due to severe diseases and serious injuries, their body’s metabolic needs have increased. On the other hand, their usual nutrition is disturbed because most are not conscious or cannot eat normally due to illness [6]. . Since the physical problems of these patients are numerous and complex, providing them with physical comfort is primarily tricky, and from the point of view of nurses, it is said to be one of the most challenging parts of end-of-life care [8, 28, 32].

#### Theme 4- psychosocial and spiritual considerations

Meeting the psychosocial and spiritual needs of the patient has been a constant part of end-of-life care education programs in many studies [3, 6, 8, 12, 13, 20–27, 29–32]. The management of emotional problems, anxiety, and depression in the patient and family is one of the essential components that was emphasized in several reviewed studies [3, 6, 12, 25]. These studies stated that dealing with end-of-life psychological problems can be debilitating for the patient and overwhelming for the family [6, 12]. In addition, most studies have emphasized that end-of-life care should be family-based while maintaining the patient’s independence as much as possible. Hence, these items were included in the educational content of the reviewed studies [20, 22, 30]. Also, spirituality was one of the other basic dimensions of some educational programs of the studied studies [3, 8, 13, 19, 25, 31, 32]. They argued that spirituality is the most personal and unknown dimension of human beings, which may change when the time of death arrives, and the nurse should be able to provide spiritual care at this critical stage [8, 31].

#### Theme 5- ethical considerations

Undoubtedly, getting to know the ethical principles of end-of-life care and facing the ethical challenges of this period is one of the most complex and sensitive parts of this care. Therefore, this dimension was considered in almost all reviewed studies [3, 8, 13, 23, 24, 29–31, 34]. According to the specific nature of the job, nurses must have a complete understanding of the four principles of biomedical ethics to provide appropriate care at the end of life by observing these principles [3, 8, 23, 30, 31, 33]. Also, sometimes, they are exposed to difficult ethical decisions at the end of life, the most important of which are advanced care planning and withholding and withdrawing treatment [3, 8, 13, 24, 29, 30, 34]. Therefore, they must be ready to face the complex ethical challenges of this period [13].

#### Theme 5- after-death care

One of the essential topics in the field of end-of-life care is after-death care, which is considered in most of the educational content of the reviewed studies [8, 21, 23, 24, 29, 32, 34]. The studies in this section emphasize the two issues of supporting the deceased person’s family and helping them get through the loss and grief with minimal damage [4, 6, 8, 12, 23, 32, 34]. The death of a family member has profound physical, psychological, emotional, social, and economic effects on other members and makes them go through the difficult stages of loss and mourning. Therefore, supporting family members is essential to end-of-life care [25, 27]. In addition, corpse care and legal considerations after death are among the topics that have been paid attention to in the educational content of some studies [3, 21].

## Discussion

This study aimed to identify the main elements of end-of-life care in nursing education programs. Based on the summary of the studies in this review, the components of end-of-life care education programs were categorized into six main axes. The first component was the need to familiarize learners with the principles of end-of-life care. Ozturk Birge et al. stated that nurses and nursing students have many questions regarding palliative care and end-of-life philosophy. They like to talk about the process of death and the subsequent mourning. Therefore, it is necessary to clarify these concepts in related educational programs [23]. Also, Cordeiro et al. stated in their study that clinical terms have administrative, clinical, and academic implications. Therefore, at the beginning of every educational program, the words and terms of that program must be clearly defined. For example, they stated that although palliative and end-of-life care are related, they are two different care areas and should be defined separately, with their differences highlighted [35]. Harden (2021) believes that accurate definitions of words and terms are necessary to prepare and organize the educational program [36]. Therefore, defining terms and stating the history and principles of end-of-life care at the beginning of related educational programs can resolve possible ambiguities and create a suitable mental base for learners and educators [11, 23].

Another main component of end-of-life care in the reviewed studies was communication skills, which, according to Wang et al., is the fundamental pillar of this care, and the nurse’s mastery of these skills is the basis for optimal care. However, Coyle et al. stated that despite the centrality of nurses in the health care team’s communication process, only some receive formal communication training, mainly related to end-of-life care [37]. It should be noted that nurses’ communication skills are essential for patient care, as they provide the bulk of care and support to patients and their families during the illness [25, 33]. Finally, Ekberg et al. stated that training and strengthening communication skills are vital in improving nurses’ participation in the end-of-life care of patients, and this critical issue should be included in educational programs [38].

The third component of the reviewed educational programs was the need to learn the physical care of the dying patient. In most studies, this section focused on pain management in patients. For example, in the End-of-Life Nursing Education Consortium (ELNEC) educational program, two areas of pain management and management of other symptoms were dedicated to examining the patient’s physical problems [8, 29]. In some educational content, this part was integrated with other parts of the program [14, 24]. Since most body systems are affected in the final stages of life, the physical needs of these patients are numerous and complex [5]. Haavisto et al. stated that although more than 90% of these patients experience severe pain at the end of life, they have other critical physical needs, such as skin, excretory, and respiratory problems that require special attention [38]. Also, in another study, Welsch et al. criticized the focus only on pain relief in dying patients and considered it against the comprehensive care approach in nursing [39].

Psychological, social, and spiritual considerations were another component of reviewing end-of-life educational programs in this study. In the psychosocial dimension, the focus of end-of-life educational programs was on managing anxiety, depression, and emotional suffering in the patient and family. In this context, Sultana et al. stated that psychosocial health is a significant concern of end-of-life care worldwide. They pointed out that patients and caregivers involved in end-of-life care may experience severe psychosocial conditions that adversely affect the quality of this care [40]. In this regard, Goode et al. also stated in their study that nurses should specialize in teaching coping skills to patients and companions so that they can manage the situation in cases of increased stress and emotional tensions [27]. On the other hand, Rosenberg et al. believed that the dying patient has emotional and psychological needs other than anxiety and depression, which should be taken care of by the nurse. They recommended that in end-of-life care, attention should be paid to the whole person instead of focusing on disease processes [41]. Providing patient-centered and family-centered care were two other dimensions of psychosocial considerations addressed in end-of-life educational programs. In this regard, O’Shea et al. emphasized that patient-centered and family-centered care is the main characteristic of a quality clinical care program and a standard care education program [30]. In most educational content reviewed, spiritual considerations were essential to end-of-life care plans. In this context, Dobrowolska et al. stated in their study that spiritual care is an inherent aspect of end-of-life care, which has been neglected in related educational programs in recent years. This issue has caused an increase in the unmet spiritual needs of these patients [25].

Ethical considerations, as the fifth component of end-of-life educational programs, were the most sensitive and challenging part of these programs. In this dimension, two issues of getting to know the ethical principles in end-of-life care and how to face the ethical challenges of this period were included in the programs. There are four universally recognized ethical principles in providing care, especially at the end of life: Autonomy, beneficence, nonmaleficence, and justice [23, 30, 31]. In this context, Akdeniz et al. stated that since decisions made may concern family and community members of patients as well as patients, it is essential to protect the rights, dignity, and power of all parties involved in the clinical ethical decision-making process. Therefore, when providing end-of-life care, nurses should be aware of and learn the internationally recognized ethical principles of care [42]. Deciding to withhold or withdraw treatment or advanced care planning are two of the most common end-of-life care challenges that cause ethical dilemmas [24, 29]. Jack et al. stated that most nurses need more confidence in their knowledge and skills in fulfilling their ethical obligations in this field and feel the need for more training [43]. Also, O’Shea et al. emphasized the consideration of laws and policies of societies and countries in the design of educational programs, especially in ethical and legal aspects [30].

Along with the above factors, after-death care was recognized as the last component of end-of-life care education programs. In the same context, Mota-Romero et al. mentioned that nurses should be prepared for the events after the patient’s death during end-of-life care and be able to manage the situation appropriately [6]. Also, Hao et al. stated that since the patient’s condition deteriorates, the patient and especially his family become involved in loss and mourning, which is the peak of this phenomenon after the patient’s death. Therefore, nurses should provide the necessary support to the family to pass this stage [4]. In the end, it should be said that legal issues, especially after the patient’s death, are essential aspects of end-of-life care, knowing that nurses can provide the necessary guidance to the deceased’s family [3].

### Strengths and limitations

One strength of this review was the use of an acknowledged framework for conducting scoping reviews, as described by Arksey and O’Malley [18] updated by Peters et al. [19]. Also, the reporting was supported by the PRISMA-ScR checklist [44].

Developing the search strategy and comprehensive search for published studies was done in close collaboration with an experienced research librarian, and the search strategy was discussed several times. However, some specialized studies relevant to end-of-life care educational programs may have yet to be considered. Also, no comprehensive judgment has yet to be made about the quality of the included studies, and the studies have been selected only in terms of access to evidence and answers to the current research question and not based on the strengths and weaknesses of the findings. Hence, any educational and clinical implications should be interpreted with caution. Also, some relevant studies may have been excluded due to language limitations.

## Conclusion

End-of-life care may be considered the most complex and sensitive type of care in nursing. This is why many nurses need to prepare to provide such care. In this review, the educational content of end-of-life care was reviewed in different studies and classified into six main components. The information obtained from this review can help nursing education and treatment managers develop more comprehensive training programs to improve the quality of end-of-life care. What the researchers felt during the study was the limitation of the studies conducted in this field despite the importance of the subject. It is hoped that more rich findings will be reported in this field in the coming years to obtain a comprehensive and accurate framework for end-of-life care.

## Data Availability

Data used in this manuscript consist of published articles which cannot be shared by the authors for copyright reasons but are available through subscription to the relevant journals/databases.
